# Paradoxical Th1 activation and CTLA-4 regulation is beneficial during latent cryptococcosis

**DOI:** 10.1128/mbio.01528-26

**Published:** 2026-07-22

**Authors:** M. Ding, J. Drnevich, J. M. Yoder, E. V. Dang, K. Nielsen

**Affiliations:** 1 Department of Microbiology and Immunology, University of Minnesota 205104, Minneapolis, Minnesota, USA; 2 Roy J. Carver Biotechnology Center, University of Illinois, Urbana-Champaign 612021, Urbana, Illinois, USA; 3 Department of Biomedical Sciences and Pathobiology, Virginia Tech 1757, Blacksburg, Virginia, USA; 4 NIAID Laboratory of Host Immunity and Microbiome, Rockville, Maryland, USA; 5 Ragon Institute, Cambridge, Massachusetts, USA; Instituto Carlos Chagas, Curitiba, Brazil

**Keywords:** CD4, T-cell activation, *Cryptococcus neoformans*, cryptococcosis, AIDS, granulomatous immune response, immune mechanisms, immune

## Abstract

*Cryptococcus neoformans* is the predominant causative agent of cryptococcal meningitis in immunocompromised individuals. Conversely, in immunocompetent individuals, *C. neoformans* establishes a latent pulmonary infection characterized by a paucity of clinical symptoms. Using a mouse inhalation model of latent *C. neoformans* infection, we previously showed that CD4 T cells are necessary for preventing fungal proliferation in the lungs. In the current study, we performed single-cell RNA sequencing (scRNA-seq) and found that the CD4 T-cell response was both highly heterogeneous and dichotomous during pulmonary *C. neoformans* infection, with concomitant expression of genes related to Th1 polarization (*Tbx21* and *Ifng*) and immune regulation (*Ctla4*). First, we demonstrated that cells with Th1-like phenotypes are necessary and sufficient to control latent infection via adoptive transfer of Tbet-positive cells into infection-matched CD4-depleted recipient mice. Second, scRNA-seq analysis revealed that the subpopulation of effector CD4 T cells that co-expressed *Ctla4* and *Gata3* was significantly higher than a subpopulation that co-expressed *Ctla4* and *Tbx21*. Furthermore, our data suggested that CTLA-4 upregulation is beneficial against *C. neoformans* infection as CTLA-4 blockade promoted fungal proliferation. Thus, we propose a model wherein Th1 control of latent *C. neoformans* infection is supported by CTLA-4 suppression of detrimental Th2 activation.

**IMPORTANCE:**

CD4 T cells are a critical part of the adaptive immune response in many infections. Upon inhalation of the fungal pathogen *Cryptococcus neoformans*, CD4 T cells are necessary to control the initial latent infection and prevent disease. In immunocompromised individuals that lack CD4 T cells, the initial infection in the lungs can spread to the brain and cause lethal meningitis. In this study, we show that the CD4 T cells present during latent *C. neoformans* infection are heterogeneous with expression of diverse polarization and immune regulation markers that function in concert to prevent disease. Our studies highlight the previously unappreciated diversity and complex regulation of the CD4 T-cell response that is required to prevent disease in this important fungal pathogen.

## INTRODUCTION

*Cryptococcus neoformans* is an opportunistic fungal pathogen that causes life-threatening cryptococcal meningitis in immunocompromised individuals. It is the second-most common advanced HIV-associated infection worldwide, with an estimated mean global cryptococcal antigenemia prevalence of 4.4% among people with CD4 counts less than 200 cells/μL (1, 2). Following the inhalation of spores or desiccated yeast cells, *C. neoformans* establishes an initial pulmonary infection that is contained within granulomas (3–5). In healthy individuals, *C. neoformans* is able to persist within the lungs in the absence of any clinical symptoms (6). However, this latent infection can disseminate from the lungs and progress to fatal cryptococcosis in immunocompromised individuals (6).

Characterization of the host immune response to latent *C. neoformans* infection is limited. The majority of our knowledge about the host response to *C. neoformans* infection is derived from mouse studies using highly virulent *C. neoformans* strains that establish lethal infections, such as KN99α (7–9) and H99 strains (10, 11). We recently developed a mouse inhalation model of latent *C. neoformans* infection, utilizing the fully virulent *C. neoformans* clinical isolate UgCl223 (12). With this mouse model, we demonstrated that depletion of the CD4 T cells resulted in disseminated lethal meningitis, and lung Tbet+ Th1 cells appeared to be the predominant canonical CD4 T-cell subset (12). Surprisingly, the proportion of activated pulmonary Th1 cells expressing IFNγ upon PMA/ionomycin restimulation is significantly decreased during latent infection compared to that of uninfected controls (12). These findings suggested that while Th1 cells are important in the host adaptive immune response to latent *C. neoformans* infection, they may be unable to promote complete clearance of the infection, likely due in part to their diminished capacity to produce IFNγ. However, it is unknown what other components of the pulmonary CD4 response contribute toward preventing fungal proliferation and mortality during latent *C. neoformans* infection.

In the current study, we utilized single-cell RNA sequencing (scRNA-seq) to characterize the heterogeneity of the effector CD4 T-cell response to latent and lethal *C. neoformans* infection. Our analysis revealed a complex network of effector CD4 T-cell subsets with seemingly antagonistic qualities. To explore these paradoxical immune responses, we parsed out the interplay between the proinflammatory Th1 response and a regulatory CTLA-4 response using adoptive transfer and antibody blockade studies to elucidate the complex role that these diverse CD4 T cells play in the control of latent *C. neoformans* infection.

## RESULTS

### Heterogeneity of CD4 T-cell responses at the single-cell level

We previously showed that CD4 T cells are necessary for controlling latent *C. neoformans* infection, with depletion of CD4 T cells resulting in a significant increase in lung and brain fungal burdens in infected mice and subsequent mortality (12). Our findings also demonstrated that Tbet-expressing Th1 cells were prevalent during latent *C. neoformans* infection (12). This contrasts with lethal *C. neoformans* infections, where detrimental Th2 cells predominate (9). Surprisingly, we identified Tbet+ and Gata3+ Th1/Th2 hybrid cells within the lungs of latently infected mice, which implied that the host response could be more heterogeneous than previously theorized (12). Thus, we utilized single-cell RNA sequencing (scRNA-seq) analysis to define the heterogeneity of the host response to latent *C. neoformans* infection and resolve the individual subsets of major immune cell populations.

We initially performed a preliminary study to examine the total lung immune response to *C. neoformans* infection. CD45+ cells were isolated from lung homogenates of a mouse latently infected with UgCl223 at 14 days post-infection (DPI), a mouse latently infected with UgCl223 at 150 DPI, a mouse lethally infected with KN99α at 14 DPI, and an uninfected mouse. Using scRNA-seq analysis, we classified the lung CD45 cells into major immune cell populations using reference-based annotation (Fig. S1A and B). T cells were the only population in which we identified a visibly dramatic shift in cluster size between the different experimental conditions (Fig. S1C). ScRNA-seq analysis of lung CD45+ cells proved very useful in understanding the overall transcriptional heterogeneity of the pulmonary immune population during *C. neoformans* infection, but using bulk CD45+ lung cells limited our ability to resolve the CD4 T-cell subpopulations during the *C. neoformans* infection (Fig. S1D). Although differential expression analysis of the CD4 T cells showed a significant change in some immunomodulatory genes (Fig. S1E), additional analysis was limited due to the low number of *Cd4-*expressing cells present in the total leukocyte populations.

**Fig 1:**
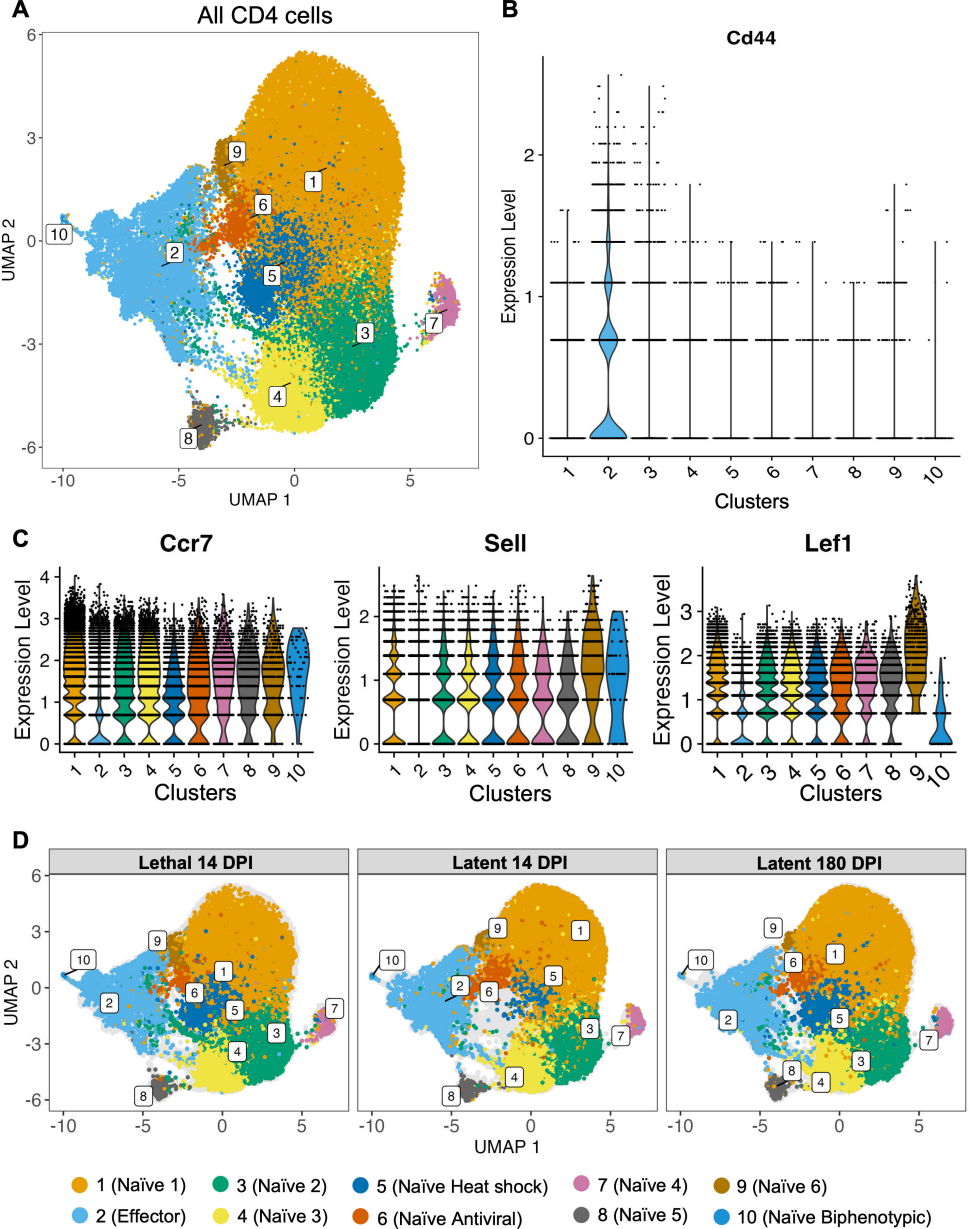
Single-cell RNA sequencing analysis of pulmonary CD4 T cells during *C. neoformans* infection. C57BL/6J mice were infected with *C. neoformans* KN99α at 14 days post-infection (Lethal 14 DPI), UgCl223 at 14 days post-infection (Latent 14 DPI), and UgCl223 at 180 days post-infection (Latent 180 DPI). CD4+ cells were isolated from lung homogenates via negative selection and processed for single-cell RNA sequencing analysis via the 10× Genomics platform. (**A**) Uniform manifold approximation and projection (UMAP) projection showing Seurat clustering of CD4 T cells. (**B**) Violin plot of *Cd44* expression (marker of T-cell activation) across all Seurat clusters. (**C**) Violin plots of *Ccr7*, *Sell*, and *Lef1* expression (markers of naïve T cells) across all Seurat clusters. (**D**) Individual UMAPs of the three experimental conditions were labeled based on the inferred subtype using the top 10 expressed genes in each Seurat cluster (Fig. S2).

To enhance the detection of the CD4 T-cell populations, we designed a more focused scRNA-seq experiment using CD4 T cells isolated via negative selection from lung homogenates of mice latently infected with UgCl223 at 14 DPI, latently infected with UgCl223 at 180 DPI, and lethally infected with KN99α at 14 DPI. Following scRNA-seq analysis, we identified 10 CD4 T-cell subsets (Fig. 1A). Only Cluster 2 included effector T cells based on their high expression of *Cd44*, a marker of T-cell activation (Fig. 1B). The remaining CD4 T-cell subsets had significantly increased expression of the naïve CD4 T-cell genes *Ccr7*, *Sell*, and *Lef1* (13, 14) (Fig. 1C). Although the top 10 expressed genes in the naïve CD4 T-cell subsets were unique (Fig. S2), many of the genes were not previously studied in the context of CD4 T cells, and this limited our ability to characterize the naïve CD4 T-cell subsets (Fig. 1D). Of note, we did observe the expression of genes involved in T-cell activation, such as *Camk1d* and *Ifit3*/*Ifit1* (Fig. S2) (15, 16), as well as a noticeable change across experimental conditions in naïve cells expressing heat shock genes (cluster 5) and the effector cell populations (cluster 2) (Fig. 1D).

**Fig 2:**
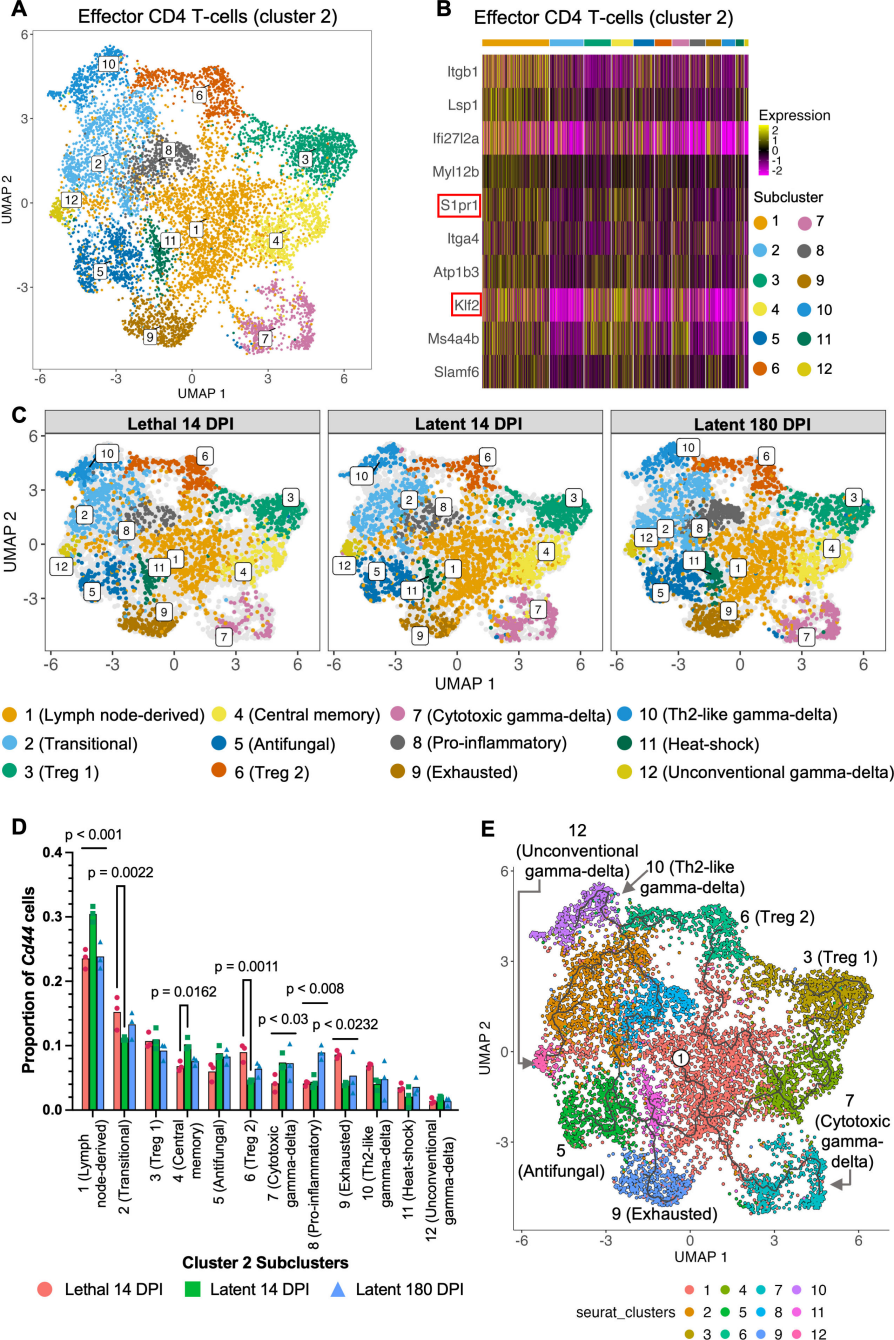
Single-cell RNA sequencing analysis of pulmonary effector CD4 T cells during *C. neoformans* infection. (**A**) UMAP projection of effector CD4 T cells (cluster 2) by Seurat clusters. (**B**) Heatmap of top 10 genes expressed by the effector CD4 T-cell cluster (cluster 2), with yellow to purple intensity indicating the mean expression of each gene and the cluster number indicated by the colored bar. (**C**) UMAP split according to the experimental condition: *C. neoformans* KN99α at 14 days post-infection (Lethal 14 DPI), UgCl223 at 14 days post-infection (Latent 14 DPI), and UgCl223 at 180 days post-infection (Latent 180 DPI) and labeled according to the inferred subtype based on the top 10 expressed markers in the Seurat cluster (Fig. S3). (**D**) Proportion of pulmonary effector CD4 T cells in the Seurat clusters across the experimental conditions: Lethal 14 DPI, Latent 14 DPI, and Latent 180 DPI. For each experimental condition, the proportion was calculated by dividing the number of cells in each effector CD4 T-cell subcluster by the total number of effector CD4 T cells. Significance was determined by two-way analysis of variance (ANOVA) with Bonferroni correction. (**E**) Branched pseudotime trajectory of effector CD4 T-cell activation/differentiation inferred by Monocle 3 with the lymph-node subcluster as the root. Each cell was colored based on Seurat clustering, and the end trajectories of the branch points were labeled.

Based on these findings, we narrowed our subsequent analyses to the *Cd44*-expressing effector CD4 T-cell subset in cluster 2. Additional clustering analysis of the *Cd44*-expressing cells (cluster 2) revealed 12 effector CD4 T-cell subclusters (Fig. 2A). The top 10 expressed genes with known functions in the effector CD4 T-cell subclusters were used for annotation (Fig. 2B; Fig. S3). Subcluster 1 was defined as lymph node-derived based on high expression of the lymph node egress genes *S1pr1* and *Klf2* (17). Subcluster 2 was defined as transitional based on high expression of chemokine receptor gene *Cxcr6* involved in mediating inflammation (18) and immune polarization genes *Rbpj*, *Ccr8*, and *Bhlhe40* (19–24). Subcluster 3 was defined as regulatory T cells (Treg 1) based on high *Foxp3* expression (25). Subcluster 4 was defined as central memory cells based on high expression of chemokine receptor gene *Ccr7* (26, 27). Subcluster 5 was defined as an antifungal effector cell cluster based on high expression of the *Myo1f* gene, which was previously implicated in antifungal immunity against *Candida albicans* and *Aspergillus fumigatus (28, 29*). Subcluster 6 was defined as a second regulatory T-cell subcluster (Treg 2) with high co-expression of *Foxp3* and *Areg* (30), along with the expression of the immune exhaustion gene *Ctla4* (31). Subcluster 7 was defined as cytotoxic gamma-delta T cells based on high expression of the cytotoxic genes *Nkg7* and *Eomes* (32, 33) and high expression of the gamma T-cell receptor gene *Trgv2* (34). Subcluster 8 was defined as a cluster of proinflammatory effector cells based on high expression of the proinflammatory genes *Ahnak* and *Rnf19b* (35–38). Subcluster 9 was defined as exhausted CD4 T cells based on high expression of exhaustion-associated genes *Tox2*, *Sostdc1*, and *Lag3* (39–41). Subcluster 10 was defined as Th2-like gamma-delta T cells based on high expression of the type-2 associated gene *Arg1* (42) and the delta and gamma T-cell receptor genes *Trdv4* and *Tcrg-C4*, respectively (34). Subcluster 11 was defined as heat shock-related CD4 T cells based on high expression of the heat shock genes *Hspa1a* and *Hspa1b*. Finally, subcluster 12 was defined as unconventional gamma-delta T cells based on high expression of the gamma-delta transcriptional regulator gene *Sox13* (43), gamma T-cell receptor gene *Tcrg-V4* (34), innate-like unconventional T-cell development regulator gene *Zbtb16 (44*), and proinflammatory IL-23 receptor gene *Il23r* (45, 46) (Fig. 2C; Fig. S3).

When examining the differences in the proportion of cells in the effector CD4 subclusters, we noted a few significant differences. Specifically, the proportion of cytotoxic gamma-delta T cells (subcluster 7) was significantly decreased and the proportion of exhausted CD4 cells (subcluster 9) was significantly increased in the lethal infection when compared to both latent infection time points (Fig. 2D). In addition, the proportion of proinflammatory effector CD4 T cells (subcluster 8) was increased during the 180 DPI latent infection, and the proportion of lymph node-derived CD4 T cells was higher during 14 DPI latent infection, compared to the other two experimental conditions (Fig. 2D). Aside from these quantitative differences, the overall UMAP projection among the three experimental conditions was similar in appearance (Fig. 2C).

The progression of effector T-cell activation and differentiation was inferred using pseudotime trajectory analysis of the subclusters. We selected the lymph node-derived subcluster (subcluster 1) as the root node. The expression of the lymph node egress genes *S1pr1* and *Klf2* (17) suggested that they were new arrivals into the lung environment. In addition, these cells also expressed the T-cell proliferation regulatory gene *Ms4a4b* (47, 48) and the T-cell activation gene *Slamf6* (49, 50) (Fig. 2B). The pseudotime analysis revealed multiple branch points, each with distinct trajectories (Fig. 2E). Notably, the majority of trajectories ended at the following clusters: Treg 1, antifungal effector cells, Treg 2, cytotoxic gamma-delta T cells, exhausted CD4 T cells, Th2-like gamma-delta T cells, and unconventional gamma-delta T cells (Fig. 2E). Overall, the pseudotime analysis demonstrated that many of the effector CD4 subsets may be precursors or intermediates during CD4 polarization.

### Increased type-1 polarization of effector CD4 T-cell responses during latent infection

**Fig 3:**
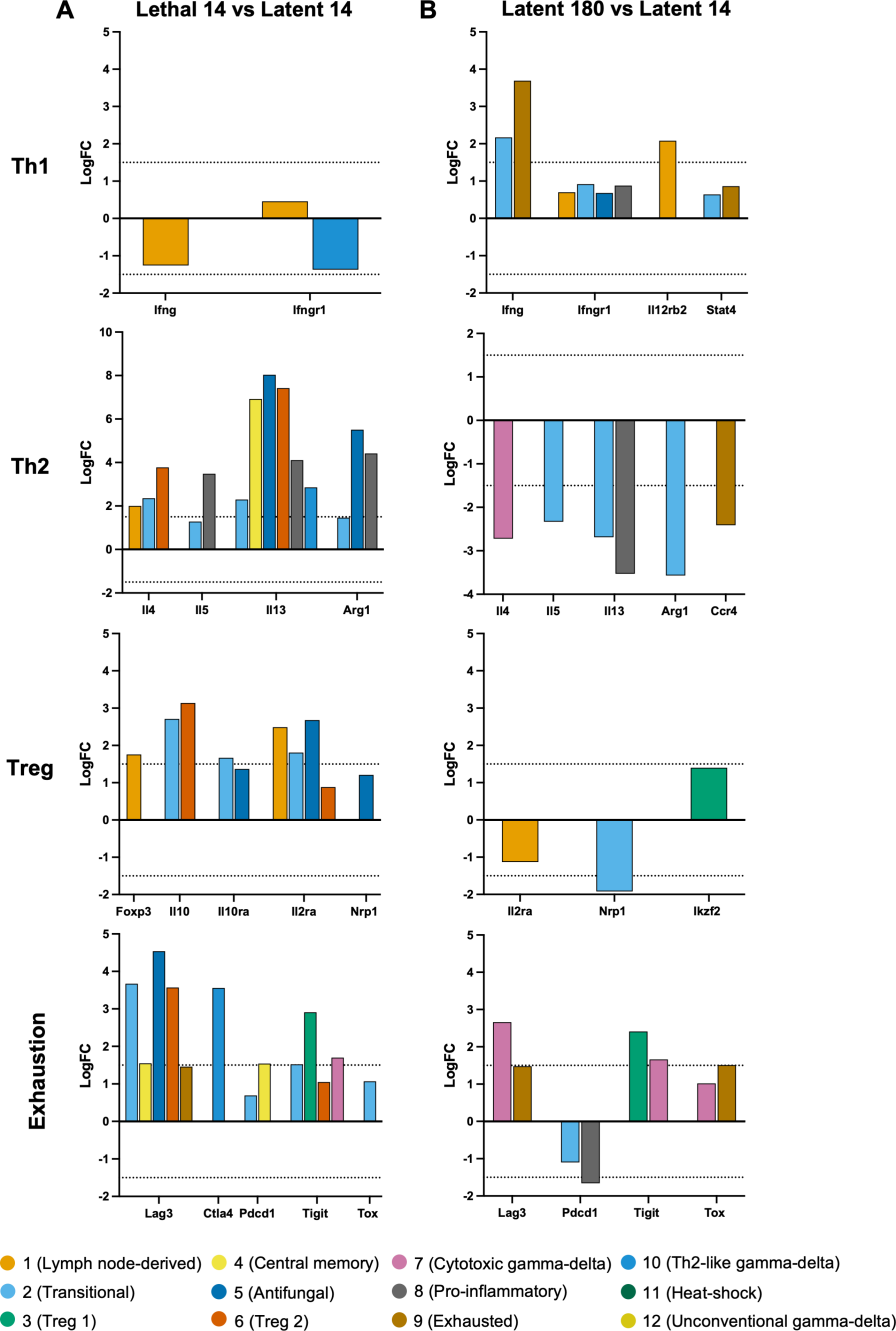
Th1-related genes are increased, and Th2-related genes are decreased over the course of latent *C. neoformans* infection. Differential expression analysis of pulmonary effector CD4 T-cell subclusters 1–12 was performed between two experimental conditions: (**A**) *C. neoformans* KN99α at 14 days post-infection (Lethal 14 DPI) and UgCl223 at 14 days post-infection (Latent 14 DPI) and (**B**) *C. neoformans* UgCl223 at 180 days post-infection (Latent 180 DPI) and Latent 14 DPI. For each comparison, significant Th1-, Th2-, Treg-, and exhaustion-associated genes within each subcluster (with globally adjusted *P*-value < 0.05) were plotted according to logFC. Positive logFC values indicate higher expression in the first condition listed, whereas negative logFC values indicate higher expression in the second condition. The dashed horizontal line denotes biological significance at |logFC| ≥ 1.5.

Transcriptional differences between the effector cell subsets during the latent and lethal infections were analyzed via differential gene expression analysis. First, we compared gene expression between the lethal infection at 14 DPI and the latent infection at the same 14 DPI time point (Fig. 3A). The differential expression analysis showed significant upregulation of the Th2-associated genes *Il4*, *Il5*, and *Il13* and the type-2-associated gene *Arg1* in the lethal infection. This was coupled with significant upregulation of the immune suppression genes *Foxp3, Il10ra, Il10*, *Il2ra,* and *Nrp1* in the lethal infection. (Fig. 3A). However, there was also significant upregulation of genes related to immune cell regulation and inhibition (*Lag3*, *Pdcd1*, *Ctla4*, and *Tigit*) in the lethal infection compared to the latent infection (Fig. 3A). Combined with the significant increase in the proportion of exhausted CD4 T cells during lethal infection, these findings indicated that the lethal *C. neoformans* infection had a robust Th2-mediated CD4 T-cell response with increased immune suppression and inhibitory gene expression when compared to the latent infection.

Second, we analyzed gene expression differences between the early latent infection 14 DPI time point compared to the late latent infection time point at 180 DPI (Fig. 3B). The early time point served as a useful control and represented an immune state where fungal burden is not yet optimally controlled, whereas the late time point was chosen to occur after the pulmonary granuloma has effectively contained and controlled the *C. neoformans* infection (51). This analysis revealed the significantly increased expression of the Th1-associated cytokine gene *Ifng* (IFNγ) over time (Fig. 3B). Interestingly, we also observed an increase in the immune inhibitory genes *Lag3* and *Tigit* in the cytotoxic gamma-delta (subcluster 7) and Treg 1 (subcluster 3) effector CD4 T-cell populations, respectively, in the late stage of the latent infection (Fig. 3B). However, we also observed a decrease in the immune-inhibitory gene *Pdcd1* in the proinflammatory (subcluster 8) effector CD4 T-cell population (Fig. 3B). Finally, there was a significant decrease in the Th17-associated gene *Il17a* (Fig. S4) and the Th2-associated genes *Il4*, *Il5*, *Il13*, *Arg1*, and *Ccr4* (Fig. 3B) in the late stage of the latent infection.

**Fig 4:**
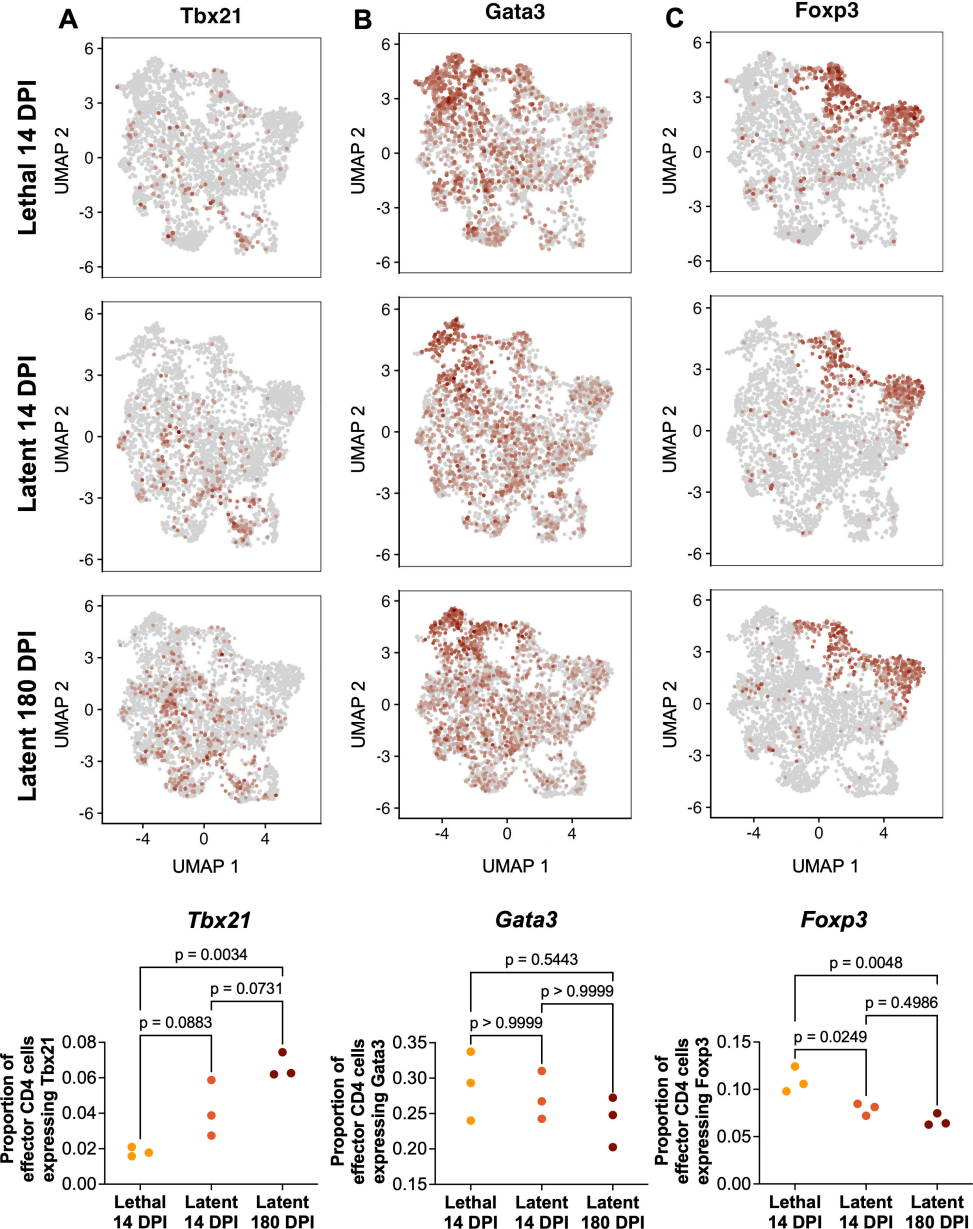
*Tbx21* (Tbet) expression by pulmonary effector CD4 T cells is significantly increased during latent *C. neoformans* infection. Feature plots showing the expression of (**A**) *Tbx21* (Tbet), (**B**) *Gata3* (GATA3), and (**C**) *Foxp3* (FoxP3) by pulmonary effector CD4 T cells across the three experimental conditions: *C. neoformans* KN99α at 14 days post-infection (Lethal 14 DPI), UgCl223 at 14 days post-infection (Latent 14 DPI), and UgCl223 at 180 days post-infection (Latent 180 DPI). Cells expressing more than 0 copies of the respective gene of interest were quantified and divided by the total number of effector pulmonary CD4 T cells to determine the proportion of effector CD4 T cells expressing the respective gene of interest in each biological replicate. Significance was determined via one-way ANOVA with Bonferroni correction.

Indeed, the proportion of all effector CD4 T cells expressing the Th1 transcription factor gene *Tbx21* was significantly increased when comparing the late latent infection (180 DPI) to lethal infection (Fig. 4A). There was also a nonsignificant trend toward a decreased proportion of effector CD4 T cells expressing the Th2 transcription factor gene *Gata3* (Fig. 4B). No significant difference in the proportion of effector CD4 T cells expressing the Th17 transcription factor gene *Rorc* or the Tfh transcription factor gene *Bcl6* was observed across all three experimental conditions (Fig. S5). However, there was a significant decrease in the proportion of effector CD4 T cells expressing the Treg transcription factor gene *Foxp3* in the latent infections compared to the lethal infection (Fig. 4C). Thus, these findings indicate an increase in Th1 polarization and a decrease in Treg polarization over time during latent *C. neoformans* infection from 14 days post-infection to 180 days post-infection.

**Fig 5:**
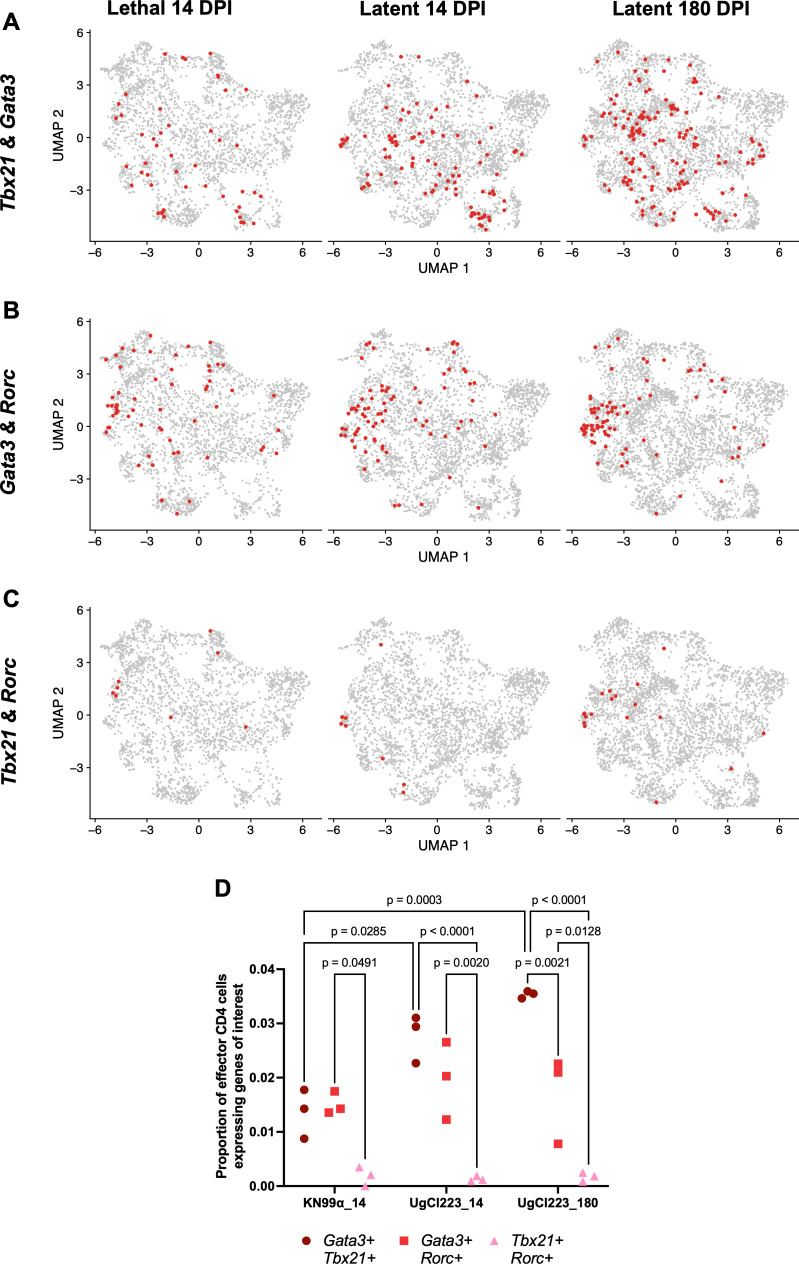
Co-expression of *Tbx21* (Tbet) and *Gata3* (GATA3) is significantly increased during latent *C. neoformans* infection. Feature plots showing co-expression of (**A**) *Tbx21* (Tbet) and *Gata3* (GATA3), (**B**) *Gata3* (GATA3) and *Rorc* (RORγt), and (**C**) *Tbx21* (Tbet) and *Rorc* (RORγt) by pulmonary effector CD4 T cells across the three experimental conditions: *C. neoformans* KN99α at 14 days post-infection (Lethal 14 DPI), UgCl223 at 14 days post-infection (Latent 14 DPI), and UgCl223 at 180 days post-infection (Latent 180 DPI). (**D**) Cells expressing more than 0 copies of the respective genes of interest were quantified and divided by the total number of effector CD4 T cells to determine the proportion of effector CD4 T-cell co-expressing the respective genes of interest in each biological replicate. Significance was determined via two-way ANOVA with Bonferroni correction.

Surprisingly, we also observed hybrid cells co-expressing multiple transcription factor genes (Fig. 5). A higher proportion of cells co-expressing *Tbx21* (Tbet) and *Gata3* (GATA3) was observed in the latent infections compared to the lethal infection (Fig. 5A and D). Cells co-expressing *Gata3* and *Rorc* (RORγt) and co-expressing *Tbx21* and *Rorc* were also identified in the effector subclusters (Fig. 5B and C), but their prevalence did not differ across the various infections (Fig. 5D).

**Fig 6:**
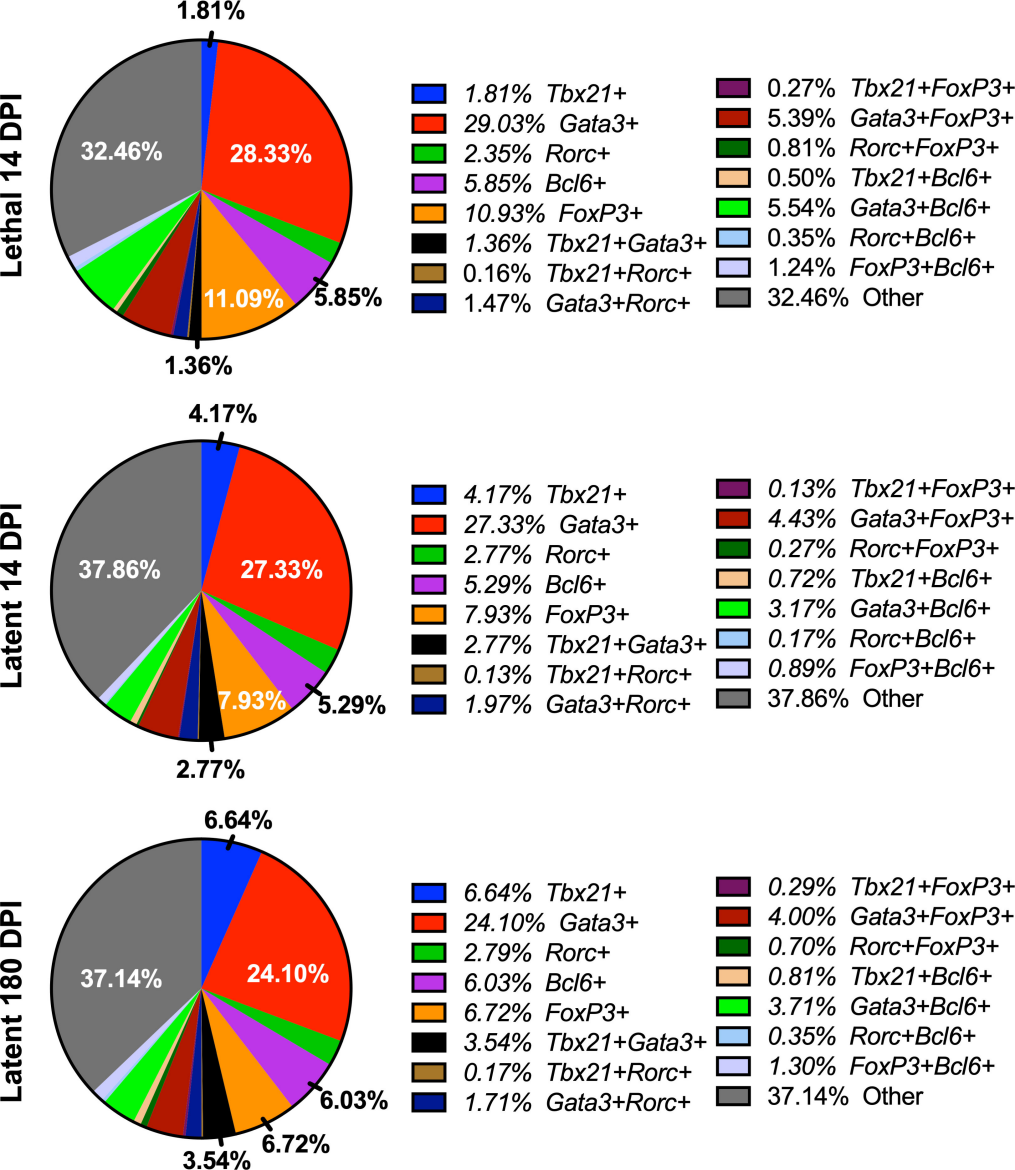
Pulmonary effector CD4 T-cell response to *C. neoformans* infection is highly heterogenous. Pie charts showing the average percentage of pulmonary effector CD4 T cells expressing lineage-defining transcription factor(s) across the three experimental conditions: *C. neoformans* KN99α at 14 days post-infection (Lethal 14 DPI), UgCl223 at 14 days post-infection (Latent 14 DPI), and UgCl223 at 180 days post-infection (Latent 180 DPI). Effector CD4 T cells expressing more than 0 copies of the transcription factor(s) of interest were identified via scRNA-seq analysis and percentage calculated from the total number of effector CD4 T cells. The remaining effector CD4 T cells without known transcription factor expression were categorized as “Other.” Refer to Table S1 for standard error calculations.

While Th1 polarization during latent infection increased from 14 to 180 DPI, there was still substantial heterogeneity at the 180 DPI time point. Cells expressing the Th1, Th2, Th17, and Tfh canonical transcription factors alone were identified (Fig. 6; Table S1). In addition, populations of hybrid cells expressing multiple transcription factors were also observed (Fig. 6). Importantly, the largest population of cells in each infection were not identifiable based on the expression of the canonical transcription factors (indicated as “other” in Fig. 6). Preliminary analysis of this “other” population revealed the population was highly heterogeneous with the expression of many genes that are not functionally characterized (data not shown). Taken together, these data not only show an upregulation of genes associated with type-1 polarization in latent infections but also highlight a lack of known gene markers to characterize the entirety of the effector CD4 response to latent *C. neoformans* infection.

### Adoptive transfer of Tbet+ CD4 T cells can control latent infection in CD4-depleted mice

**Fig 7:**
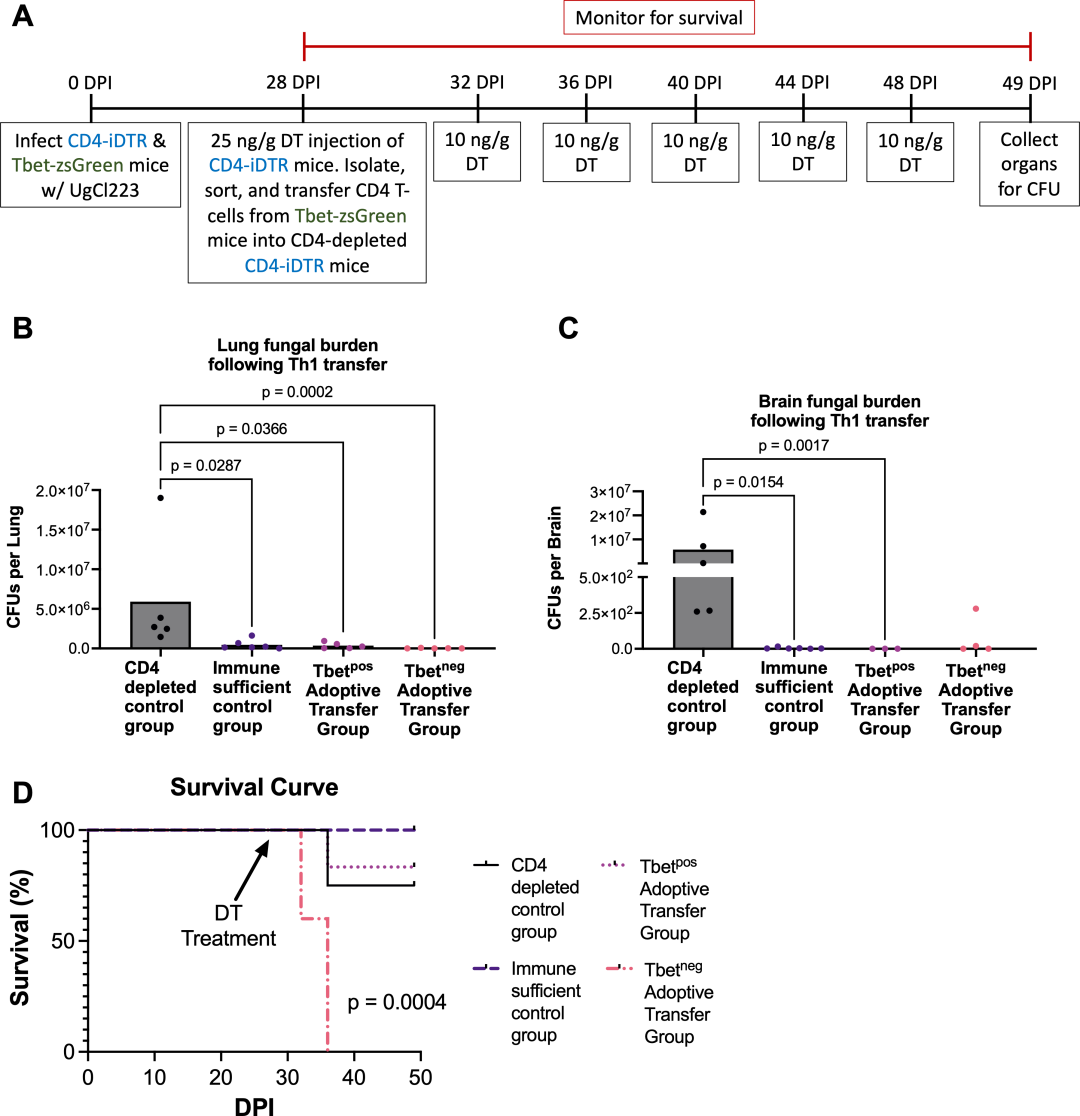
The pulmonary Th1-like response can control latent infection. (**A**) Tbet-zsGreen FoxP3-RFP mice and CD4-iDTR mice were latently infected with *C. neoformans* UgCl223. At 28 days post-infection (DPI), CD4-iDTR mice were injected with 25 ng/g diphtheria toxin (DT) via intraperitoneal (IP) injection. On the same day, the lungs were isolated from infection-matched Tbet-zsGreen FoxP3-RFP mice, digested, negatively selected for CD4 T cells, sorted for high zsGreen expression (Tbet^pos^ Adoptive Transfer Group) and low zsGreen expression (Tbet^neg^ Adoptive Transfer Group), and injected into the DT-treated CD4-iDTR mice. Every 4 days, the Tbet^pos^ Adoptive Transfer Group, Tbet^neg^ Adoptive Transfer Group, CD4-iDTR mice that were not injected with Tbet-zsGreen CD4 T cells (CD4-depleted control group), and iDTR (immune-sufficient control group) mice received a maintenance dose of 10 ng/g DT via IP injection. At 49 DPI, the control and Tbet^pos^ adoptive transfer groups were euthanized, and (**B**) the lungs and (**C**) brain of mice were harvested, homogenized, and plated for enumeration of colony-forming units (CFUs). The Tbet^neg^ adoptive transfer group mice were euthanized, and the lungs and brain were harvested at the terminal endpoint (36 DPI). Significance was determined by one-tailed nonparametric *t*-test. (**D**) Mice were monitored for signs of morbidity and euthanized at the terminal endpoint (20% total weight loss, 1 g/day weight loss for 2 consecutive days, or neurological symptoms including loss of sternal recumbency, partial paralysis, seizure, convulsion, or coma). Kaplan-Meier survival kinetics and significance were analyzed using log-rank testing.

Given the enhanced Th1-like polarization observed during latent infection, we hypothesized that cells expressing the Th1 transcription factor *Tbet* are critical for controlling the latent infection. To isolate the effects of Tbet-expressing CD4 cells, lung-resident Tbet-zsGreen-positive FoxP3-RFP-negative CD4 cells were isolated from the lungs of Tbet-zsGreen FoxP3-RFP mice latently infected with UgCl223 and then transferred into infection-matched CD4-depleted CD4-iDTR mice (henceforth referred to as “Tbet^pos^ adoptive transfer group”) and assessed for fungal burden (Fig. 7A). Our results showed that the Tbet^pos^ adoptive transfer group had equivalent lung and brain fungal burden to the iDTR control mice (henceforth referred to as the “immune sufficient control group”) and had significantly lower lung and brain fungal burden when compared to the DT-treated CD4-iDTR mice latently infected with UgCl223, which did not receive any CD4 cells (henceforth referred to as “CD4-depleted control group”) (Fig. 7B and C). Furthermore, 83% of the Tbet^pos^ adoptive transfer group survived until 49 days post-infection, compared to 75% of the CD4-depleted control group and 100% of the immune-sufficient control group (Fig. 7D). Overall, these data demonstrated that the Tbet-expressing CD4 T cells were sufficient to control latent *C. neoformans* infection in mice.

To examine the effects of CD4 T cells that did not express Tbet, we transferred lung resident Tbet-zsGreen-negative FoxP3-RFP-negative CD4 T cells isolated from the lungs of Tbet-zsGreen FoxP3-RFP mice latently infected with UgCl223 into infection-matched CD4-depleted CD4-iDTR mice (henceforth referred to as the “Tbet^neg^ adoptive transfer group”). Surprisingly, the Tbet^neg^ adoptive transfer group had 0% survival at 36 days post-infection (Fig. 7D). The terminal lung and brain fungal burden was measured at this 36 DPI endpoint, prior to the 49 DPI time point used for the other mouse populations. Based on the previous studies, we would anticipate no significant difference in fungal burden across the 36 and 49 DPI time points; and there was no difference in lung and brain fungal burden of the Tbet^neg^ adoptive transfer group compared to the Tbet^pos^ adoptive transfer group and the immune-sufficient control group (Fig. 7B and C). Instead, we observed a significant decrease in lung fungal burden in the Tbet^neg^ adoptive transfer group compared to the CD4-depleted control group (Fig. 7B). These data suggest that, while the Tbet^neg^ cell population was able to control *C. neoformans* growth, these cells were unable to prevent mortality.

### IFNγ signaling is necessary for control of latent *C. neoformans* infection

**Fig 8:**
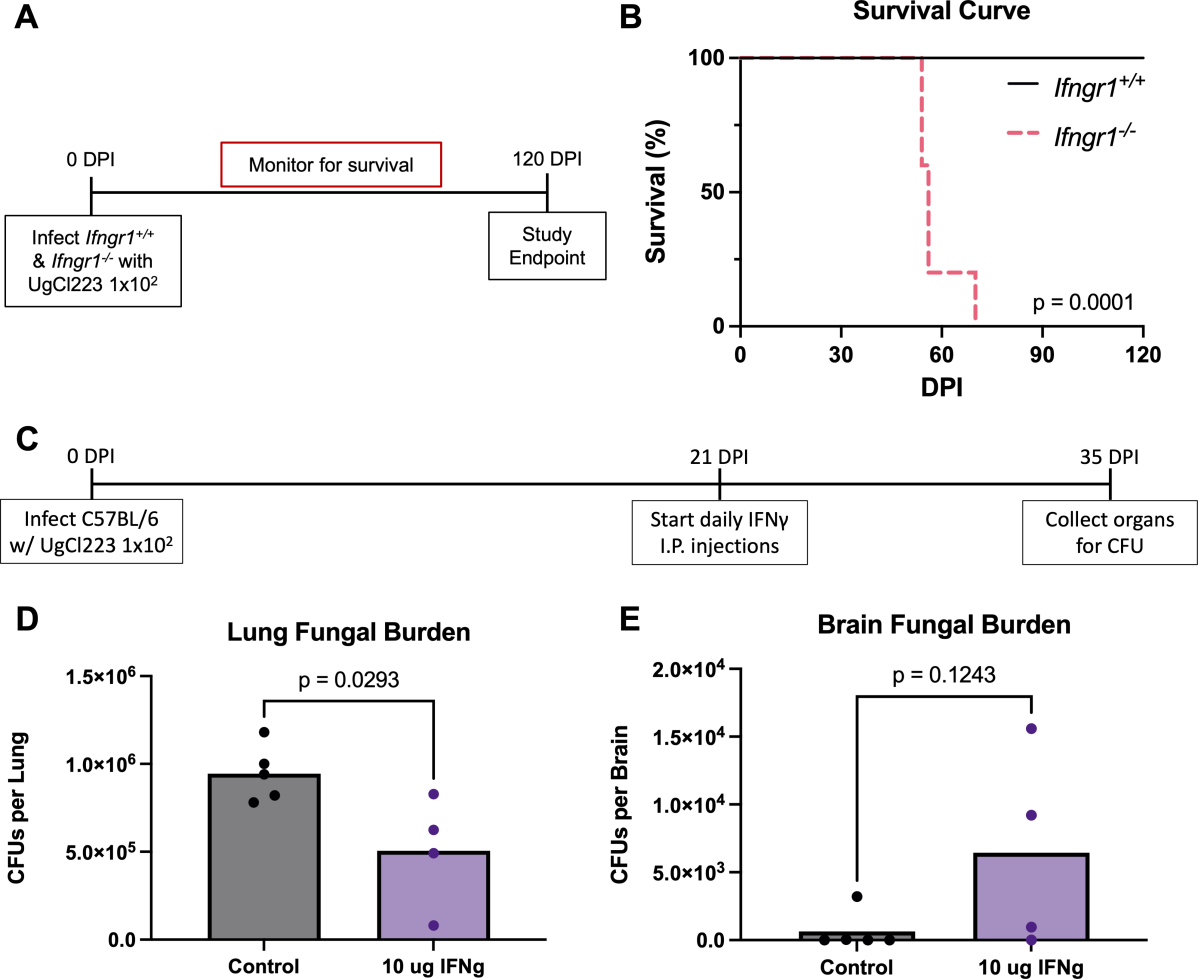
Exogenous IFNγ supplementation decreases lung fungal burden during latent *C. neoformans* infection. (**A**) *Ifngr1^+/+^* (i.e., C57BL/6J) and *Ifngr1^−/^*^−^ mice were latently infected with *C. neoformans* UgCl223. (**B**) Mice were monitored for signs of morbidity and euthanized at the terminal endpoint (20% total weight loss, 1 g/day weight loss for 2 consecutive days, or neurological symptoms including loss of sternal recumbency, partial paralysis, seizure, convulsion, or coma). Kaplan-Meier survival kinetics and significance was analyzed using log-rank testing. (**C**) C57BL/6J mice were infected with *C. neoformans* UgCl223. At 21 days post-infection (DPI), mice were injected with 10 μg IFNγ or sterile PBS vehicle control via weekly intraperitoneal injections. At 35 DPI, (**D**) the lungs and (**E**) brain of mice were harvested, homogenized, and plated for enumeration of colony-forming units (CFUs). Significance was determined by two-tailed *t*-test.

Having demonstrated that Tbet+ cells were necessary and sufficient for controlling latent infection, we next aimed to clarify whether the downstream Th1-associated pathways, particularly IFNγ signaling, were similarly essential. *Ifngr1^+/+^* and *Ifngr1^−/^*^−^ mice were latently infected with UgCl223 and monitored for survival (Fig. 8A). We observed 100% mortality of the latently infected *Ifngr1^−/^*^−^ mice, with a median survival of 56 DPI (Fig. 8B). These findings indicate that IFNγ signaling is a necessary component of the Th1 inflammatory response.

Interestingly, our previous study noted that pulmonary CD4 T cells isolated from latently infected mice had reduced IFNγ production when compared to cells isolated from uninfected control mice (12). We hypothesized that this diminished IFNγ production by the Tbet+ CD4 T cells during latent infection prevents complete clearance of the infection and posited that exogenous IFNγ supplementation would enhance the antifungal response. To test this hypothesis, mice were latently infected with UgCl223 and injected daily with 10 μg of mouse recombinant IFNγ starting at 21 DPI. At 35 DPI (14 days post-IFNγ treatment), lung and brain fungal burden was assessed (Fig. 8C). While the lung fungal burden in the IFNγ-treated mice was significantly decreased compared to that of sterile saline-treated control mice, treatment with 10 μg was not sufficient to completely clear the infection from the lungs (Fig. 8D). In addition, IFNγ treatment resulted in a nonsignificant trend toward increased brain fungal burden (*P*-value = 0.1243) (Fig. 8D). Thus, IFNγ supplementation reduced lung fungal burden during latent infection but did not completely resolve the infection.

### CTLA-4 expression was significantly increased during latent *C. neoformans* infection

**Fig 9:**
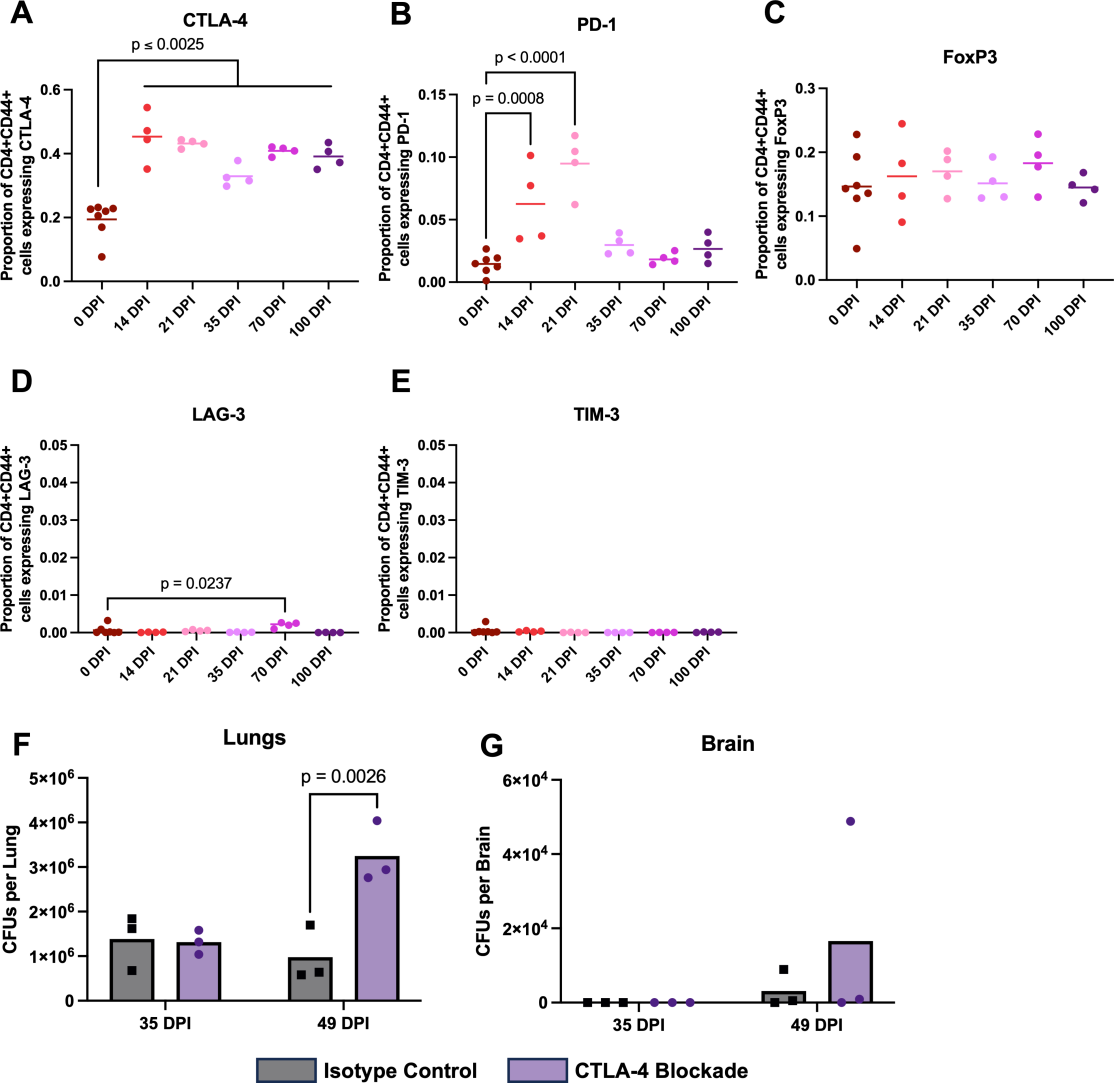
CTLA-4 blockade increases lung fungal burden. At 0, 14, 21, 35, and 100 days post-infection, the lungs of C57BL/6J mice infected with *C. neoformans* UgCl223 were isolated and digested into single-cell suspensions, and the proportion of (**A**) CTLA-4+, (**B**) PD-1+, (**C**) LAG3+, (**D**) TIM3+, and (**E**) FoxP3+ expressing CD4+ CD44+ cells were quantified via flow cytometry based on the total number of lung CD4+ CD44+ cells. Significance was determined by one-way ANOVA with Bonferroni correction. For CTLA-4 blockade, C57BL/6J mice were infected with *C. neoformans* UgCl223 and at 21 days post-infection (DPI), and mice were injected with 0.5 mg anti-CTLA4 monoclonal antibody (9D9) or IgG2b isotype control via weekly intraperitoneal injections. At 35 and 49 days post-infection, (**F**) the lungs and (**G**) brain of mice were harvested, homogenized, and plated for enumeration of colony-forming units (CFUs). Significance was determined by two-way ANOVA with Bonferroni correction.

Although we clearly established that Th1 polarization was beneficial in controlling pulmonary *C. neoformans* infection, we sought to reconcile this finding with the prominent inhibitory gene expression observed in our scRNA-seq analysis of effector CD4 cells (cluster 2) (Fig. S2). Indeed, the proportion of effector CD4 cells expressing *Ctla4* was significantly increased compared to all other known regulators of immune exhaustion regardless of the experimental condition (Fig. S6). The predominance of *Ctla4* expression was also reflected in our flow cytometry data, where the proportion of activated CD4+ CD44+ FoxP3 T cells producing CTLA-4 was significantly increased throughout the latent infection, in comparison to cells isolated from uninfected mice (Fig. 9A). In contrast, this phenomenon was not observed in activated CD4+ CD44+ FoxP3 T cells producing PD-1, FoxP3, LAG-3, or TIM-3 (Fig. 9B through E). Overall, both the scRNA-seq and flow cytometry analysis suggested that *Ctla4* (CTLA-4) could be integral to immunomodulation during latent infection, albeit with unclear implications.

**Fig 10:**
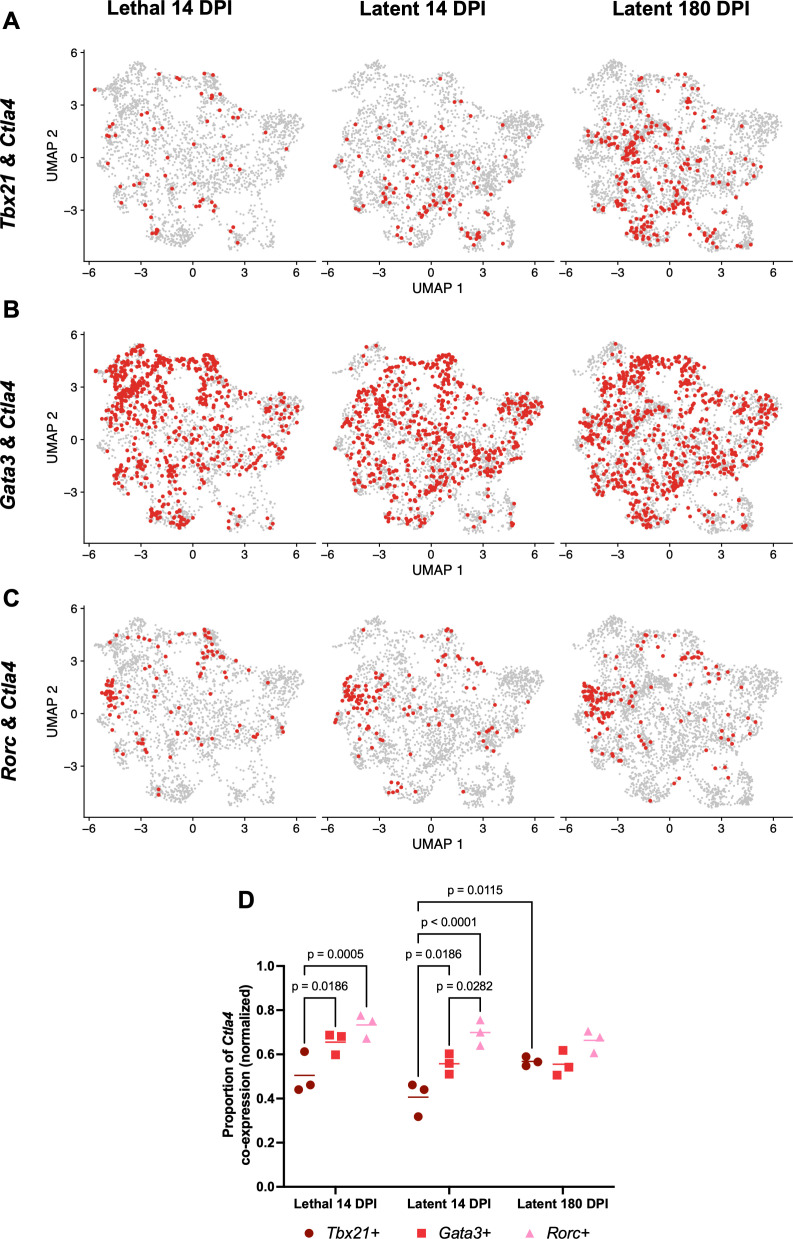
Co-expression of *Ctla4* (CTLA-4) and *Gata3* (GATA3) is significantly increased during *C. neoformans* infection. Feature plots showing co-expression of (**A**) *Tbx21* (Tbet) and *Ctla4* (CTLA-4), (**B**) *Gata3* (GATA3) and *Ctla4* (CTLA-4), and (**C**) *Rorc* (RORγt) and *Ctla4* (CTLA-4) by pulmonary effector CD4 T cells across the three experimental conditions: *C. neoformans* KN99α at 14 days post-infection (Lethal 14 DPI), UgCl223 at 14 days post-infection (Latent 14 DPI), and UgCl223 at 180 days post-infection (Latent 180 DPI). (**D**) Effector CD4 T cells co-expressing more than 0 copies of *Ctla4* and more than 0 copies of *Tbx21*, *Gata3*, or *Rorc* were quantified and divided by the total number of cells expressing *Tbx21*, *Gata3*, or *Rorc*, respectively, to determine the proportion of each cell population with *Ctla4* co-expression in each biological replicate. Significance was determined via two-way ANOVA with Bonferroni correction.

Intriguingly, within the scRNA-seq data, there were differences in the expression of *Ctla4* among the effector CD4 cells that expressed either *Tbx21* (Th1), *Gata3* (Th2), or *Rorc* (Th17) across all experimental conditions (Fig. 10A through C). For both lethal and latent infections at 14 DPI, the proportion of *Tbx21*-expressing effector CD4 T cells that also expressed *Ctla4* was significantly lower than the proportion of *Gata3*-expressing and *Rorc*-expressing effector CD4 T cells with *Ctla4* co-expression (Fig. 10D). The only significant difference between the experimental conditions was an increase in the proportion of *Tbx21 Ctla4* co*-*expression between 14 DPI and 180 DPI (Fig. 9D). These data indicate that *Ctla4* is more highly expressed by Th2 and Th17 cells, rather than Th1 cells, in early latent and lethal infections.

To determine the role of CTLA-4 during latent infection, we tested whether blockade of CTLA-4 influenced fungal proliferation in the latent infection model. Following latent infection with UgCl223, mice were injected weekly with anti-CTLA-4 monoclonal antibody starting at 21 DPI. At 35 and 49 DPI, lung and brain fungal burden were assessed. CTLA-4 blockade promoted fungal proliferation, with significantly higher lung fungal burden in mice treated with the CTLA-4 monoclonal antibody compared to mice treated with an isotype control at 49 DPI (Fig. 9F). We also observed a nonsignificant increase in brain fungal burden between the CTLA-4 blockade and the isotype control mice (Fig. 9G). Altogether, these data support a protective role for CTLA-4 in the context of early latent *C. neoformans* infection.

## DISCUSSION

CD4 T cells are a necessary host defense against *C. neoformans* infection, as evidenced by the significant global burden on HIV-associated cryptococcal meningitis (1). These observations are also well corroborated by *in vivo* animal depletion studies that used monoclonal antibodies (52, 53) and diphtheria toxin-induced cell lineage ablation (12), which showed that CD4 T cells are necessary for pulmonary control of *C. neoformans* infection. In this study, we further characterized the effector CD4 T-cell response via scRNA-seq analysis and identified key mechanisms of host control against latent *C. neoformans* infection.

Using scRNA-seq, we explored the transcriptional landscape of the CD4 T-cell response against both lethal and latent *C. neoformans* infections. We identified 10 distinct CD4 T-cell subsets, of which nine were naïve CD4 T cells, and the remaining cluster was annotated as effector CD4 T cells (cluster 2) based on the upregulation of *Cd44*. Notably, the top 10 expressed genes for each naïve CD4 T-cell cluster were unique to each individual cluster, which highlighted the heterogeneity of the pulmonary naïve CD4 response. The expression of genes involved in T-cell activation such as *Camk1d* and *Ifit3*/*Ifit1* also suggested that the naïve CD4 T-cell repertoire was influenced by the *C. neoformans* infection (15, 16).

Following the identification of the effector CD4 cluster, we performed additional clustering analysis and revealed 12 effector CD4 subclusters. As with the naïve CD4 T-cell clusters, annotation of the effector CD4 subclusters was limited to well-characterized genes. However, by using pseudotime analysis, we were able to further infer the trajectories of CD4 T-cell activation and differentiation from the “newly arrived” lymph node-derived CD4 T cells. This pseudotime analysis, when combined with the differential gene expression analysis of the effector CD4 subclusters, revealed a significant increase in Th1 polarization and decrease in Th2 and Th17 polarization over the time course of the latent infection. These findings are consistent with those of our previous studies that showed that the immune response during latent infection changes from a preliminary Th2 and Th17 response to the mature Th1 response (51). Thus, we now have strong evidence that links latent *C. neoformans* infection with increased Th1 polarization.

Importantly, the scRNA-seq analysis of pulmonary CD4 T cells during *C. neoformans* infection highlighted the immense transcriptional heterogeneity of the CD4 T-cell response and revealed that a substantial proportion (~30%) of effector CD4 T cells could not be identified based on known transcription factor production. These cells express various poorly characterized genes; thus, it remains unclear what role these undefined CD4 T cells play during latent *C. neoformans* infection. The identification of hybrid cell populations is also intriguing and suggests more nuanced regulation of the phenotype of Th cells than previously appreciated. Importantly, the diversity among the CD4 T cells we observed is predominantly within the “Lung CD4+ TD” population in the newly proposed T-cell nomenclature and highlights the challenges related to classifying the diversity of CD4+ T-cell phenotypes during infection and identification of key cell populations (54, 55).

By comparing (i) lethal and latent infections at the same time point and (ii) early- and late-latent infection time points, we gained a better understanding of the Th cell phenotypes that influence disease outcomes in *C. neoformans*. Lethal infections promoted a strong Th2 response with significant upregulation of immunosuppressive regulatory genes in the majority of effector subclusters. These data are consistent with those of previous studies showing that Th2 responses dominate during lethal *C. neoformans* infections (8, 9). Importantly, the significant increase in the proportion of effector CD4 T cells expressing *Tbx21*, or co-expressing *Tbx21* and *Gata3*, during latent infection corroborates the damage-response framework theory (56–58), where the host response to *C. neoformans* infection oscillates between a beneficial controlled type-1 polarization versus a detrimental type-2 polarization.

Surprisingly, the Tbet+ Th1-like CD4 T cells only account for 10% of the total effector cell population. Yet the adoptive transfer of pulmonary Tbet+ CD4 cells proved that Tbet+ CD4 T cells are necessary and sufficient to control latent *C. neoformans* infection. Of note, this experimental set-up was not able to differentiate between CD4 T cells that only express Tbet+ and hybrid cells that co-express Tbet+ and other lineage-defining transcription factors. Regardless, the significant decrease in fungal burden and improved survival of the Tbet^pos^ adoptive transfer group compared to the CD4-depleted group suggests that the diverse Tbet+ CD4 populations were both necessary and sufficient for preventing proliferation and dissemination and ultimately mortality during *C. neoformans* latent infection.

With regard to the Tbet^neg^ adoptive transfer group, the low fungal burden observed during this experiment suggests that the Tbet^neg^ CD4 cells were at least partially capable of controlling the fungal infection. We cannot rule out the possibility that this decreased fungal burden simply reflects the earlier time point (36 DPI) of organ collection compared to the other experimental and control groups (49 DPI); however, previous latent infection studies show no significant changes in fungal burden between 36 and 49 DPI (12, 51). However, the increased mortality of mice receiving the Tbet^neg^ adoptive transfer is striking and suggests that these cells could act as a double-edged sword, helping control fungal proliferation yet eliciting rapid mortality. Of note, the design of the adoptive transfer experiments excluded Tregs, which may be critical for dampening or controlling the activity of the diverse Tbet^neg^ effector CD4 T-cell subsets. It is also conceivable that the beneficial activities of the undefined CD4 T-cell subsets were masked by the function of Gata3+ Th2-like CD4 T cells, which were previously shown to induce detrimental type-2 responses during lethal infections (9).

In addition to demonstrating that Tbet+ cells are necessary and sufficient for controlling latent infection, we also showed that downstream IFNγ signaling is a key component, with 100% of latently infected *Ifngr1^−/^*^−^ mice succumbing to the infection. Furthermore, we observed a decrease in lung fungal burden with exogenous IFNγ treatment in latently infected mice. However, IFNγ supplementation did not completely clear latent infection, in contrast to previous studies where infection with a *C. neoformans* strain genetically modified to produce IFNγ resolved without additional interventions (59, 60). It is certainly possible that the diminished IFNγ response observed during latent infection (12, 61) may reflect *C. neoformans*-mediated modulation of the host response and long-term persistence within the lungs, but our findings reflect the difficulties in determining an appropriate therapeutic dose for IFNγ. These conclusions are not surprising as adjunctive IFNγ therapy did not impact survival outcomes between treated and untreated patient cohorts, although a decrease in brain fungal burden was observed in these human clinical trials (62). While a higher dose of IFNγ may theoretically result in greater fungal clearance, the trend toward increased brain fungal burden with IFNγ supplementation that we observed suggests that IFNγ may enhance either dissemination or growth of *C. neoformans* in the brain via a phenomenon similar to immune reconstitution inflammatory syndrome (IRIS) (63). Thus, CD4 T-cell production of IFNγ is important for controlling *C. neoformans* infection, but its utility as a therapeutic agent may be limited.

To date, the scientific community has focused on understanding the effects of Th1 polarization on *C. neoformans* infection. However, our scRNA-seq analysis of the effector CD4 T-cell response to *C. neoformans* infection revealed upregulation of many immune regulatory genes, most significant of which was *Ctla4* (CTLA-4) (64, 65). Indeed, significant upregulation of CTLA-4 was observed via flow cytometry as early as 14 DPI during latent *C. neoformans* infection, which reflects previously published data demonstrating that the capsule of *C. neoformans* strains can promote rapid expression of CTLA-4 in CD4 T cells within 24 h of co-incubation (66).

Interestingly, we found that the proportion of *Gata3*-expressing Th2-like cells co-expressing *Ctla4* was significantly higher than that of the *Tbx21*-expressing Th1-like cells. Furthermore, CTLA-4 monoclonal antibody (mAb) blockade resulted in significantly increased lung fungal burden. These findings are in contrast to those of a previous study that demonstrated that CTLA-4 blockade promotes fungal clearance during lethal *C. neoformans* infection (67). The opposing experimental outcomes may be due to multiple experimental factors, including the type of *C. neoformans* strain, timing of the administration of the CTLA-4 mAb, and different inbred mouse strains. In addition, differences in host CD4 polarization against lethal and latent *C. neoformans* infection likely also contributed to differences in fungal clearance or proliferation. Regardless, our data suggest that CTLA-4 mAb blockade starting at 21 DPI enhanced fungal proliferation and lead us to hypothesize that the blockade removed CTLA-4 suppression of the detrimental Th2 effector function.

There are still some points of uncertainty regarding CTLA-4 function during *C. neoformans* infections. For instance, we observed a significant increase in the proportion of *Ctla4* expression in Th1 cells (expressing *Tbx21*) from 14 to 180 DPI during latent infection, but the implication of this finding is unclear given that there was no difference in Ctla4 expression across the cell types at 180 DPI. In addition, while Th17 cells make up a small proportion of effector CD4 T cells during *C. neoformans* infection, the proportion of *Ctla4* expression in Th17 cells (expressing *Rorc*) was significantly higher compared to that of Th1 and Th2 cells during lethal and early latent infections. These data suggest that studies to better delineate the role of Th17 cells are warranted, especially during latent infection.

In summary, we demonstrated that the CD4 T-cell response generated against *C. neoformans* infection is highly complex and heterogeneous. We definitively showed that Tbet+ cells are necessary and sufficient to control latent *C. neoformans* infection. Our findings also shed light on the nuances that drive the host response to latent versus lethal *C. neoformans* infection, with special consideration to the integral role of immune regulation. We propose that the beneficial host response that leads to control during latent *C. neoformans* infections is paradoxical—involving both accumulation of beneficial Tbet+ cells and a simultaneous CTLA-4-mediated suppression to dampen the activity of other cell types. Our data and previous studies suggest targeting IFNγ may not be a viable clinical option. However, CTLA-4 activation has not been explored and could be a novel immunomodulatory mechanism to prevent cryptococcosis.

## MATERIALS AND METHODS

### Inbred mouse strains

All mice used in this study were C57BL/6J or derived from a C57BL/6J background. Mice were housed in specific pathogen-free conditions. CD4-Cre (68) and homozygous iDTR (69) breeding pairs were purchased from Jackson Laboratory and were crossed to generate CD4-Cre/iDTR, referred to as CD4-iDTR, mice (69). Systemic CD4 T-cell ablation in the CD4-iDTR mice was achieved by an initial intraperitoneal injection of 25 ng/g diphtheria toxin (DT; Sigma) followed by an additional 10 ng/g DT every 4 days. CD4+ T cell levels were monitored to confirm the effectiveness of the DT treatment throughout the 49 DPI treatment regimen. Tbet-zsGreen mice (70) were crossed with FoxP3-RFP mice (71) to generate Tbet-zsGreen FoxP3-RFP mice and were kindly gifted by Marc Jenkins. *Ifngr1^−/^*^−^ mice were obtained from Jackson Laboratory (JAX 025,545) (72). Mice used for all experiments were 6–8 weeks of age; all controls were sex- and age-matched.

### *Cryptococcus* strains

*Cryptococcus neoformans* strains KN99α (73) and UgCl223 (74, 75) were stored as −80°C glycerol stocks, streaked on yeast peptone dextrose (YPD) +0.04 g/L chloramphenicol agar plates and incubated for 2 days at 30°C prior to use. YPD broth was inoculated with colonies from the aforementioned plate and incubated for 16 h at 30°C and 225 RPM. The resulting inoculum was prepared by centrifuging the culture for 1 min at 14,000 RPM (17,968 × *g*) to pellet the cells, washing the cells three times with phosphate-buffered saline (PBS), and resuspending the cells in PBS at a concentration of either 1 × 10^6^ cells/mL (KN99α) for lethal infections or 2 × 10^3^ cells/mL (UgCl223) for latent infections.

### Infection

A well-established intranasal pulmonary inhalation model of cryptococcosis was used for this study (73, 76). Mice were fully anesthetized with pentobarbital until they did not respond to a toe pinch. The mice were then lethally infected with 5 × 10^4^ KN99α or latently infected with 1 × 10^2^ UgCl223 *C. neoformans* cells in 50 µL of PBS by placing the inoculum on the nares of each mouse. The mice were then suspended by their incisors for 5 min and subsequently paced upright on a paper towel in their cage until regaining consciousness, enabling the inoculum to reach the lower respiratory tract.

All mice were monitored for morbidity and sacrificed at predetermined time points or when endpoint criteria were reached. Endpoint criteria were defined as 20% total body weight loss, loss of 2 g of weight in 2 days, or symptoms of neurological disease.

### Organ fungal burden

Lungs and brain were collected at the time of mouse euthanasia and placed in 2 mL sterile PBS. The collected tissues were homogenized, and serial dilutions of tissue homogenates were plated on YPD agar plates supplemented with 0.04 mg/mL chloramphenicol and incubated at 30°C. *C. neoformans* colonies were counted after 48 h of incubation.

### Single-cell RNA sequencing workflow and analysis

We conducted two separate single-cell RNA sequencing experiments. (i) A preliminary study analyzing total lung CD45+ leukocytes isolated from a mouse infected with *C. neoformans* UgCl223 at 14 days post-infection, a mouse infected with UgCl223 at 150 days post-infection, a mouse infected with *C. neoformans* KN99α at 14 days post-infection, and an uninfected mouse. (ii) A robust study analyzing lung CD4+ lymphocytes isolated from three mice infected with UgCl223 at 14 days post-infection, three mice infected with UgCl223 at 180 days post-infection, and three mice infected with KN99α at 14 days post-infection.

For both studies, the lungs were perfused with cold PBS and digested into a single-cell homogenate with collagenase A. For the CD45+ preliminary study, CD45+ cells were isolated via positive selection using the EasySep Mouse CD45 Positive Selection Kit (StemCell) as per the manufacturer’s instructions. For the CD4+ cell study, lymphocytes were first isolated via a 67%/40% Percoll density gradient, and then CD4 T cells were isolated via negative selection using the EasySep Mouse CD4+ T cell Isolation Kit (StemCell) (see “Flow cytometry methods” below for a more detailed description).

For both studies, single-cell RNA sequencing libraries were prepared using a Chromium Single Cell Controller (10× Genomics). The CD45+ preliminary study used the Chromium Single Cell 3′ Library and Gel Bead v3.1 dual index kit (10× Genomics). The CD4+ cell study used the Chromium Single Cell 5′ Library and Gel Bead v2 dual index kit (10× Genomics). Briefly, for both studies, cell suspensions were diluted in nuclease-free water to obtain a target cell recovery of 10,000 cells, according to the manufacturer’s instructions. Remaining steps were also carried out according to the manufacturer’s instructions for cDNA amplification and sample index PCR. For both studies, final libraries were sequenced on two NovaSeq 6000 (Illumina) 150-bp paired-end flow cells at the University of Illinois at Urbana-Champaign DNA Services Lab. Libraries were sequenced with read lengths of 28 × 10 × 10 × 150. For both studies, the *Mus musculus* assembly mm10-2020-A (GRCm38) from NCBI was used with Cell Ranger 7.0.1 to make a reference for alignment. The “cellranger count” command was then used to call cells and count UMIs for each gene and cell.

For both studies, UMI count matrices were imported into R from the Cell Ranger output. The CD45+ preliminary study was performed with R (version 4.0.4) and Seurat (version 4.3.0.1). The CD4+ study was performed with R (version 4.4.0) and Seurat (version 5.0.3). Genes were discarded if there were fewer than 10 cells with UMIs (unique reads). Counts were then imported into a Seurat object. Percent mitochondrial UMIs (“percent.mt”) were calculated using the 13 mitochondrial genes in the Cell Ranger reference. The percentage mitochondrial UMIs, along with total UMIs per cell and total features per cell with UMIs, were recorded and are available upon request. Cells with fewer than 200 features with UMIs were removed as they were assumed to be red blood cells, low quality, or dying cells. Cells with more than three median absolute deviations above the median number of UMIs were filtered, given that they were likely to represent doublets. Cells with more than three median absolute deviations above the median number of mitochondrial UMIs were discarded because they were assumed to be dying. UMI counts were normalized via the SCTransform method (77) using the top 3,000 most variable features and regressing on percentage mitochondrial UMIs and cell cycle scores.

For the CD45+ preliminary study, all samples were integrated using an anchor-based canonical correlation analysis (CCA) approach (78). Principal component analysis (PCA) was performed on the transformed UMI counts, and the first 40 axes were selected by the heuristic elbow method and were retained for clustering and uniform manifold approximation and projection (UMAP). Following UMAP visualization, calculation of k-nearest neighbors was performed. Clustering analysis was used to determine the appropriate resolution and number of clusters for each experimental condition (79–81). Seurat was used for identifying cell clusters (82). Microarray data from the Immunological Genome Project (83), distributed with the celldex R package (84), was used. Low-quality assignments were pruned according to default settings. Following the annotation of each Seurat cluster, differential expression analysis was performed by grouping the infected experimental conditions and comparing against the uninfected condition via a model-based analysis of single-cell transcriptomics (MAST) approach (zero-inflated negative binomial model) (85). Genes with adjusted *P*-values < 0.05 for each comparison were ranked by log2FC.

For the CD4+ study, we merged all experimental groups together but opted against performing integration across the different replicates and experimental groups since we did not observe condition-specific clustering in our merged data set (Fig. 1D). Normalization, PCA, UMAP, and clustering analysis were performed as described above. To increase the rigor of our study, we identified clusters with elevated *Cd4* expression and excluded the clusters with low expression of *Cd4* and cell clusters with non T-cell markers (i.e., endothelial, alveolar, and fibroblasts). In addition, to reduce artificial cluster separation caused by *Trbv* gene overrepresentation, all *Trbv* genes were removed from the variable gene set and all subsequent sub-clusterings. Normalization, dimensionality reduction, and clustering analysis were repeated to identify all *Cd4*-positive cell clusters. Cluster-specific markers were identified using FindAllMarkers(). Clusters were annotated based on the top 10 expressed genes. Of the 10 Cd4 clusters, only cluster 2 had high expression of *Cd44* (Fig. 1B), identifying it as the Effector Cd4 cluster.

Cluster 2 (effector) was isolated from the larger *Cd4*-positive data set for subclustering analysis, which included further normalization, dimensionality reduction, and clustering analysis. We opted to perform integration analysis during the subclustering analysis for better alignment of the cluster 2 (effector) subclusters. Differential expression analysis of the cluster 2 (effector) subclusters was performed using muscat and limma. A model matrix was constructed to compare conditions pairwise (Lethal 14 DPI versus Latent 14 DPI; Latent 180 DPI versus Latent 14 DPI). Trajectory analysis was conducted using Monocle3 (v1.3.1). Cells were ordered in pseudotime, with the trajectory root manually inferred from subcluster 1.

All sequencing data were deposited at NCBI under accession number PRJNA1271018, and the bioinformatic analysis data were deposited in Dryad at https://doi.org/10.5061/dryad.j3tx95xsx.

### Flow cytometry

We utilized intravascular staining to discriminate between vascular and tissue leukocytes as described previously (86). Briefly, mice were intravenously injected via the tail vein with APC-eFluor780-labeled CD45 antibody (30-F11, eBioscience) and were euthanized 3 min following injection with CO_2_. Pulmonary leukocytes were isolated as described previously (87). Briefly, the chest cavity was opened, cold PBS was used to perfuse lungs via the right ventricle, and the trachea was exposed. The lungs were inflated with 2 mL of digestion solution containing 1.5 mg/mL collagenase A (Roche), 5 mM DNase I (Ambion), 5% fetal bovine serum (FBS), and 10 mM HEPES (MP Biomedicals) in Hanks' balanced salt solution (HBSS; Gibco). The lungs were excised and placed in 5 mL digestion solution. Lung tissue plus digestion solution was incubated in a 37°C water bath for 30 min with gentle vortexing every 8–10 min. Upon completion of digestion, the resulting cell suspensions were strained through a 70-μm cell strainer and washed with 25 mL of PBS. Cells were then centrifuged for 10 min at 300 × *g* for 10 min at 4°C.

For CD4 T-cell isolation, the resulting cell pellet was resuspended in 40% Percoll-RPMI medium (GE Life Sciences). A Percoll density gradient was created (5 mL of 40% Percoll-RPMI top and 3 mL 67% Percoll-PBS bottom), and the samples were centrifuged for 20 min at 650 × *g* without braking. The lymphocytes at the interface were removed, washed twice with PBS containing 1 mg/mL bovine serum albumin (BSA; Sigma) and 0.002 mM EDTA (Invitrogen), and then centrifuged for 10 min at 300 × *g* for 10 min at 4°C. CD4 T cells were isolated via negative selection using the EasySep Mouse CD4+ T cell Isolation Kit (StemCell).

All single-cell suspensions were stained with Near-IR Live Dead viability dye (Biolegend) according to the manufacturer’s instructions and then incubated for 15 min on ice with CD16/32 antibody (Biolegend) to prevent nonspecific antibody binding. For surface staining, samples were stained with fluorophore-labeled antibodies at 4°C for 30 min.

For intracellular staining, cells were washed, fixed, and permeabilized using the Foxp3/Transcription Factor Staining Buffer Set (eBioscience) according to the manufacturer’s instructions. Samples were stained intracellularly with fluorophore-labeled antibodies at 4°C overnight. After staining, cells were washed with 1× permeabilization buffer, placed in cell staining buffer, and stored at 4°C until ready for data acquisition by flow cytometry.

All data were acquired with a BD LSR Fortessa flow cytometer using BD FACSDiva software (BD Bioscience). Compensation was performed at the beginning of each experiment with UltraComp eBeads plus Compensation Beads (Invitrogen) and the ArC Amine Reactive Compensation Bead Kit (Life Technologies). Data were analyzed using FlowJo v10.8.1.

For CD4 exhaustion marker identification, the following fluorophore-labeled antibodies were used: B220 (RA3-6B2, APC-eFluor780, Thermo Fisher Scientific), CD11c (N418, APC-eFluor780, Thermo Fisher Scientific), CD11b (M1/70, APC-eFluor780, Thermo Fisher Scientific), F4/80 (BM8, APC- eFluor780, Thermo Fisher Scientific), CD3 (17A2, BV650, Biolegend), CD4 (GK1.5, BUV39, BD Biosciences), CD8 (53-6.7, AF700, Thermo Fisher Scientific), CD44 (IM7, PerCp-Cy5.5, Biolegend), PD-1 (J43, APC, Invitrogen), LAG-3 (C9B7W, BV421, Biolegend), TIM-3 (RMT3-23, BV711, Biolegend), CTLA-4 (UC10-4B9, BV605, Biolegend), and FoxP3 (FJK-16S, AF488, eBioscience). The gating strategy is shown in Fig. S7.

### Adoptive transfer

Tbet-zsGreen FoxP3-RFP mice and CD4-iDTR mice were infected with *C. neoformans* UgCl223, as described above. At 28 days post-infection, CD4-iDTR mice were injected with 25 ng/g diphtheria toxin via intraperitoneal injection. On the same day, Tbet-zsGreen FoxP3-RFP mice were injected with CD45-APC via the tail vein to differentiate between organ-specific and vascular cells and euthanized after 3 min. The lungs were perfused with cold PBS and digested via collagenase A into a single-cell suspension (as described above) and stained for CD3 (17A2, AF700, Biolegend). Lung cells from 10 Tbet-zsGreen FoxP3-RFP mice were pooled together. Following negative CD4 isolation, cells were sorted for viability, and the population of cells that were (i) CD45-APC− (lung-resident), CD3-AF700+, FoxP3-RFP−, and Tbet-zsGreen+ and (ii) CD45-APC− (lung-resident), CD3-AF700+, FoxP3-RFP−, and Tbet-zsGreen− were isolated. The sorted lung resident Tbet-zsGreen+ and lung resident Tbet-zsGreen− cells were then injected into DT-treated CD4-iDTR mice (Tbet^pos^ adoptive transfer group and Tbet^neg^ adoptive transfer group, respectively). Every 4 days, the Tbet^pos^ adoptive transfer group (*n* = 6), the Tbet^neg^ adoptive transfer group (*n* = 5), CD4-iDTR mice that were not injected with Tbet-zsGreen CD4 T cells (CD4-depleted control group) (*n* = 8), and iDTR mice (immune-sufficient control group) (*n* = 6) received a maintenance dose of 10 ng/g DT via IP injection. At 49 DPI, surviving experimental and control groups were euthanized. The lungs and brains were assessed for fungal burden, as described above.

### Ifngr1*^−/^*^−^ survival curve and exogenous IFNγ supplementation

*Ifngr1^−/^*^−^ (*n* = 5) and *Ifngr1^+/+^* (i.e., C57BL/6J) (*n* = 5) mice were infected with UgCl223, as described above. Survival was monitored based on the aforementioned end-point criteria.

C57BL/6J mice were infected with UgCl223, as described above. At 21 days post-infection, mice were intraperitoneally injected with 10 μg recombinant mouse IFNγ (Biolegend) every 24 h (*n* = 4). Sterile saline was used as the vehicle control (*n* = 5). Lung and brain fungal burden was measured at 35 days post-infection, as described above.

### CTLA-4 monoclonal antibody blockade

Mice were intranasally infected with UgCl223 and intraperitoneally injected with 0.5 mg of anti-CTLA-4 monoclonal antibody (9D9; Bio X Cell) starting at 21 days post-infection and every 7 days thereafter. Because CTLA-4 blockade does not result in depletion of CTLA-4+ cells, the efficacy was assessed by flow cytometric analysis via FoxP3+ CD4 T-cell depletion (88, 89) (Fig. S8). An IgG2b (MPC-11; Bio X Cell) isotype antibody was used as a control. Lung and brain fungal burden was measured at 35 and 49 days post-infection, as described above. The following fluorophore-labeled antibodies were used for this experiment: CD45 for IV label (30-F11, APC-eFluor780, eBioscience), CD45 (30-F11, BUV805, BD Biosciences), B220 (RA3-6B2, BV650, Biolegend), CD11c (N418, APC-eFluor780, Thermo Fisher Scientific), CD11b (M1/70, APC-eFluor780, Thermo Fisher Scientific), F4/80 (BM8, APC-eFluor780, Thermo Fisher Scientific), TCRβ (H57-597, PE, eBioscience), CD4 (GK1.5, BUV39, BD Biosciences), CD8 (53-6.7, AF700, Thermo Fisher Scientific), CD44 (IM7, PerCp-Cy5.5, Biolegend), PD-1 (J43, APC, Invitrogen), FoxP3 (FJK-16S, AF488, eBioscience), and CTLA-4 (UC10-4B9, BV605, Biolegend).

### Statistics

Statistical analysis was performed with GraphPad Prism 9 software (La Jolla, CA). Power calculations were performed to assess appropriate sample size for all experiments. Data were analyzed using one- or two-tailed *t*-test or one-way or two-way ANOVA. Bonferroni adjustment for multiple comparisons was implemented when justified. *P*-values ≤0.05 were considered statistically significant. All data presented in this study, except in Fig. S1, S7, and S8Fig. S8, are representative of a minimum of at least three independent experiments or biological replicates.

## Data Availability

The data underlying Fig. 1 to 5 and 8, Fig. S1 to S6, and Table S1 are openly available in NCBI SRA under BioProject PRJNA1271018. The accompanying downstream analysis is available in Dryad: https://doi.org/10.5061/dryad.j3tx95xsx. The data underlying Fig. 6, 7, and 9, and Fig. S7 and S8 are available upon request.

## SUPPLEMENTAL MATERIAL

Figure S1 mbio.01528-26-s0001.tif Single-cell RNA sequencing analysis of pulmonary CD45 T cells during *C. neoformans* infection.

Figure S2 mbio.01528-26-s0002.tif Top 10 expressed genes per Seurat cluster of the pulmonary CD4 T-cell response to *C. neoformans* infection.

Figure S3 mbio.01528-26-s0003.tif Top 10 expressed genes per Seurat cluster of the pulmonary effector CD4 T-cell response to *C. neoformans* infection.

Figure S4 mbio.01528-26-s0004.tif Th17-related genes are decreased over the course of latent *C. neoformans* infection.

Figure S5 mbio.01528-26-s0005.tif *Rorc* and *Bcl6* expression by pulmonary effector CD4 T cells is not significantly changed during *C. neoformans* infection.

Figure S6 mbio.01528-26-s0006.tif Ctla4 expression by pulmonary effector CD4 T cells is significantly increased during *C. neoformans* infection.

Figure S7 mbio.01528-26-s0007.tif Exhaustion marker flow cytometry gating strategy.

Figure S8 mbio.01528-26-s0008.tif CTLA4 monoclonal antibody depletes FoxP3+ regulatory CD4 T cells.

Table S1 mbio.01528-26-s0009.pdf Standard error of the average percentage of pulmonary effector CD4 T cells expressing lineage-defining transcription factors.

ASM does not own the copyrights to Supplemental Material that may be linked to, or accessed through, an article. The authors have granted ASM a non-exclusive, world-wide license to publish the Supplemental Material files. Please contact the corresponding author directly for reuse.
