# Supplementary material for: Paradoxical Th1 activation and CTLA-4 regulation is beneficial during latent cryptococcosis
Source: mBio. 2026 Jul 22;17(8):e01528-26. doi: 10.1128/mbio.01528-26 (PMC13463883; doi:10.1128/mbio.01528-26)
Supplement: Supplementary file 1 [file mbio.01528-26-s0009.pdf]

	Lethal 14 DPI	Latent 14 DPI	Latent 180 DPI
**Tbx21+**	0.00151947	0.00915751	0.00405026
**Gata3+**	0.02813803	0.01967831	0.02043572
**Rorc+**	0.00493156	0.00345377	0.00583715
**Bcl6+**	0.00387	0.0094276	0.00889033
**FoxP3+**	0.00780265	0.00378834	0.0038173
**Tbx21+Gata3+**	0.00262237	0.00255136	0.00037738
**Tbx21+Rorc+**	0.00101568	0.00028466	0.00047576
**Gata3+Rorc+**	0.00120278	0.00412474	0.00467895
**Tbx21+FoxP3+**	0.00042285	0.0008244	0.00070974
**Gata3+FoxP3+**	0.00462844	0.00597748	0.00057255
**Rorc+FoxP3+**	0.00079646	0.000151	0.00063664
**Tbx21+Bcl6+**	0.00160654	0.0017423	0.00033112
**Gata3+Bcl6+**	0.00758535	0.00423198	0.00140763
**Rorc+Bcl6+**	0.00300774	0.00111927	0.00088548
**FoxP3+Bcl6+**	0.0029978	0.00034268	0.00259049
**Other**	0.02868598	0.01155426	0.01701077

Supplementary Table 1: Standard error of average percentage of pulmonary effector CD4 T-cells expressing lineage-defining transcription factor(s).
